# Concentric ring optical traps for orbital rotation of particles

**DOI:** 10.1515/nanoph-2023-0600

**Published:** 2023-11-23

**Authors:** Xing Li, Dan Dan, Xianghua Yu, Yuan Zhou, Yanan Zhang, Wenyu Gao, Manman Li, Xiaohao Xu, Shaohui Yan, Baoli Yao

**Affiliations:** State Key Laboratory of Transient Optics and Photonics, Xi’an Institute of Optics and Precision Mechanics, Chinese Academy of Sciences, Xi’an 710119, China; University of Chinese Academy of Sciences, Beijing 101408, China; Collaborative Innovation Center of Extreme Optics, Shanxi University, Taiyuan 030006, China

**Keywords:** optical tweezers, perfect optical vortices, computer generated hologram, complex amplitude modulation

## Abstract

Optical vortices (OVs), as eigenmodes of optical orbital angular momentum, have been widely used in particle micro-manipulation. Recently, perfect optical vortices (POVs), a subclass of OVs, are gaining increasing interest and becoming an indispensable tool in optical trapping due to their unique property of topological charge-independent vortex radius. Here, we expand the concept of POVs by proposing concentric ring optical traps (CROTs) and apply them to trapping and rotating particles. A CROT consists of a series of concentric rings, each being a vortex whose radius and topological charge can be controlled independently with respect to the other rings. Quantitative results show that the generated CROTs have weak sidelobes, good uniformity, and relatively high diffraction efficiency. In experiments, CROTs are observed to trap multiple dielectric particles simultaneously on different rings and rotate these particles with the direction and speed of rotation depending on the topological charge sign and value of each individual ring. In addition, gold particles are observed to be trapped and rotate in the dark region between two bright rings. As a novel tool, CROTs may find potential applications in fields like optical manipulation and microfluidic viscosity measurements.

## 1 Introduction

Optical tweezers, due to the ability to manipulate micro-objects on the scale of nano- to micro-meter, have a wide range of applications in fields like cell biology, molecular biology, colloid physics, atomic physics, and polymer material science [1–10]. The optical manipulation is, in essential, a manifestation of transferring optical linear and angular momentum arising in light-matter interaction. The former, linear momentum, can be used to trap and transport particles. The latter, especially orbital angular momentum (OAM) is usually employed to yield rotation of the trapped object.

The OAM of light is closely related to the helix phase front of light beams, namely optical vortices (OVs). In 1992, Allen et al. [11] first pointed out that Laguerre-Gaussian (LG) beams, due to their phase factor exp(*ilφ*), have a well-defined OAM equal to *lℏ* per photon, where *φ* is the azimuthal angle and *l* is the topological charge. Importantly, they recognized that the OAM could be removed from the beam and transformed into a mechanical moment of the object, which opened up a new horizon for optical manipulation of microscopic objects. In 1995, He et al. [12] observed experimentally that absorbing copper oxide (CuO) particles in water were trapped in the dark center of a LG beam, and always spun in the same direction at a constant speed. This spin rotation is induced by the OAM transfer due to the fact that the dark center size of the linearly polarized LG mode used in the experiment is comparable to the particle diameter. In 2000, O’Neil et al. [13] successfully observed the orbital rotation of silver particles driven by the OAM of a LG beam. Since then, there have been an increasing number of studies on rotating microscopic objects using the OAM of light [14–21].

OVs usually exhibit an intensity structure featuring a bright ring and a dark center. When used in optical manipulation, the gradient force is capable of trapping particles on the ring, while the scattering force leads the particles to rotate along the ring. Changing the sign of the topological charge *l* can reverse the direction of rotation. For a conventional OV, its radius of ring increases with increasing the absolute value of topological charge. However, in many applications such as particle trapping and manipulation, it is desirable to have an OV with both a small ring radius and a large topological charge. To this end, the concept of perfect optical vortices (POVs) was introduced [22, 23], whose ring radius is independent of topological charge. Such a property has inspired strong interest among researchers [24–28].

More recently, double-ring perfect optical vortices (DR-POVs), featuring two closely spaced bright rings, were created with different methods, such as the binary Dammann grating or polarization modulation. Yu et al. [29] generated line-polarized DR-POVs by using circular Dammann gratings. Liang et al. [30] obtained orthogonally circularly polarized DR-POVs by the Fourier transform of azimuthally polarized Bessel beams. Karpeev et al. [31] combined a circular Dammann grating with a polarization converter to obtain radially polarized DR-POVs. In parallel with these works on DR-POVs, Rickenstorff et al. [32] quantitatively analyzed the relationship between the ring-peak distance and the input field polarization under both paraxial and tight focusing conditions. It was found that the minimum value of the ring-peak distance is significantly affected by the input field polarization. However, when the ring-peak distance is increased to more than twice the diffraction limit, polarization effect becomes negligible. In addition, Du et al. [33] designed DR-POVs with multiple adjustable parameters, and experimentally generated them with high quality and diffraction efficiency. The above efforts have generally focused on the generation and characterization of DR-POVs and have barely involved their applications, especially in the scenario of optical trapping.

Here, we expand the concept of POVs by proposing concentric ring optical traps (CROTs), in which the number of rings is no longer limited to one or two, and explore their applications in particle trapping and orbital rotation. The CROTs exhibit a concentric-ring intensity distribution, and the radius and topological charge of each ring can be controlled separately with respect to the other rings. We obtain the complex amplitude in the holographic plane by directly superposing a series of higher-order Bessel functions with helical phase exp(*ilφ*), followed by a complex amplitude encoding, a direct method simpler than iterative methods. We quantitatively analyze the generated CROTs in terms of energy ratio, uniformity and diffraction efficiency. The results show that they are of high quality, i.e., weak sidelobes, good uniformity, and relatively high diffraction efficiency. We demonstrate theoretically and experimentally CROTs with multiple topological charge combinations and utilize them to manipulate polystyrene (PS) microspheres. As expected, the PS spheres are observed to be trapped on different rings and to orbit along these rings. In addition, as a special case of CROTs, a tunable DR-POV is used to trap and rotate gold (Au) particles that, in contrast to dielectric transparent particles, execute orbital motion in the intermediate dark zone between the two bright rings. CROTs are ideally suited to study the orbital rotation of trapped particles and might be a useful tool in microfluidic viscosity measurements.

## 2 Methods

### 2.1 Generation of CROTs

Usually, a POV has an electric field amplitude in the focal plane characterized by a ring of radius *R* and the topological charge *l*

(1) $$\begin{array}{c} E\left(r,\theta \right)=\delta \left(r-R\right)\exp\left(il\theta \right) \end{array}$$

where *δ*(*) is the Dirac delta function and (*r*, *θ*) are the polar coordinates in the focal plane. Assuming that the focal length of a lens is *f* and the wavelength is *λ*, the field *E*(*ρ*, *φ*) in the back focal plane of the lens is given by the inverse Fourier transform of Eq. (1):

(2) $$\begin{array}{ll} E\left(\rho ,\varphi \right) & =\frac{1}{\lambda f}\iint rE\left(r,\theta \right)\exp\left[i\frac{2\pi }{\lambda f}\rho rcos\left(\theta -\varphi \right)\right]\text{d}r\text{d}\theta \\ & =i^{l}\alpha J_{l}\left(\alpha \rho \right)exp\left(il\varphi \right), \end{array}$$

where (*ρ*, *φ*) are the polar coordinates in the back focal plane, *J*_*l*_(*) is an *l*-order Bessel function of the first kind, and *α* = 2π*R*/(*λf*). It is worth noting that the field *E*(*ρ*, *φ*) in the back focal plane has a topological charge exactly equal to that of the field *E*(*r*, *θ*) in the focal plane, and the radius of the vortex ring, controlled by the parameter *α*, is independent of the topological charge *l*.

By combining a set of POVs of the form Eq. (1) with different radii and topology charges, one can construct a new type of POV traps, called concentric ring optical traps (CROTs), with the field given by

(3) $$\begin{array}{c} E\left(r,\theta \right)=\overset{N}{\underset{n=1}{\sum }}\delta \left(r-R_{n}\right)\exp\left(il_{n}\theta \right), \end{array}$$

where *R*_*n*_ and *l*_*n*_ denote the radius and topological charge of the *n*th ring, and *N* denotes the total number of rings. Similarly, the field *E*(*ρ*, *φ*) in the back focal plane is given by

(4) $$\begin{array}{c} E\left(\rho ,\varphi \right)=\overset{N}{\underset{n=1}{\sum }}i^{l_{n}}\alpha_{n}J_{l_{n}}\left(\alpha_{n}\rho \right)\exp\left(il_{n}\varphi \right), \end{array}$$

where *α*_*n*_ = 2π*R*_*n*_/(*λf*) determines the radius of the *n*th ring.

**Figure 1:**
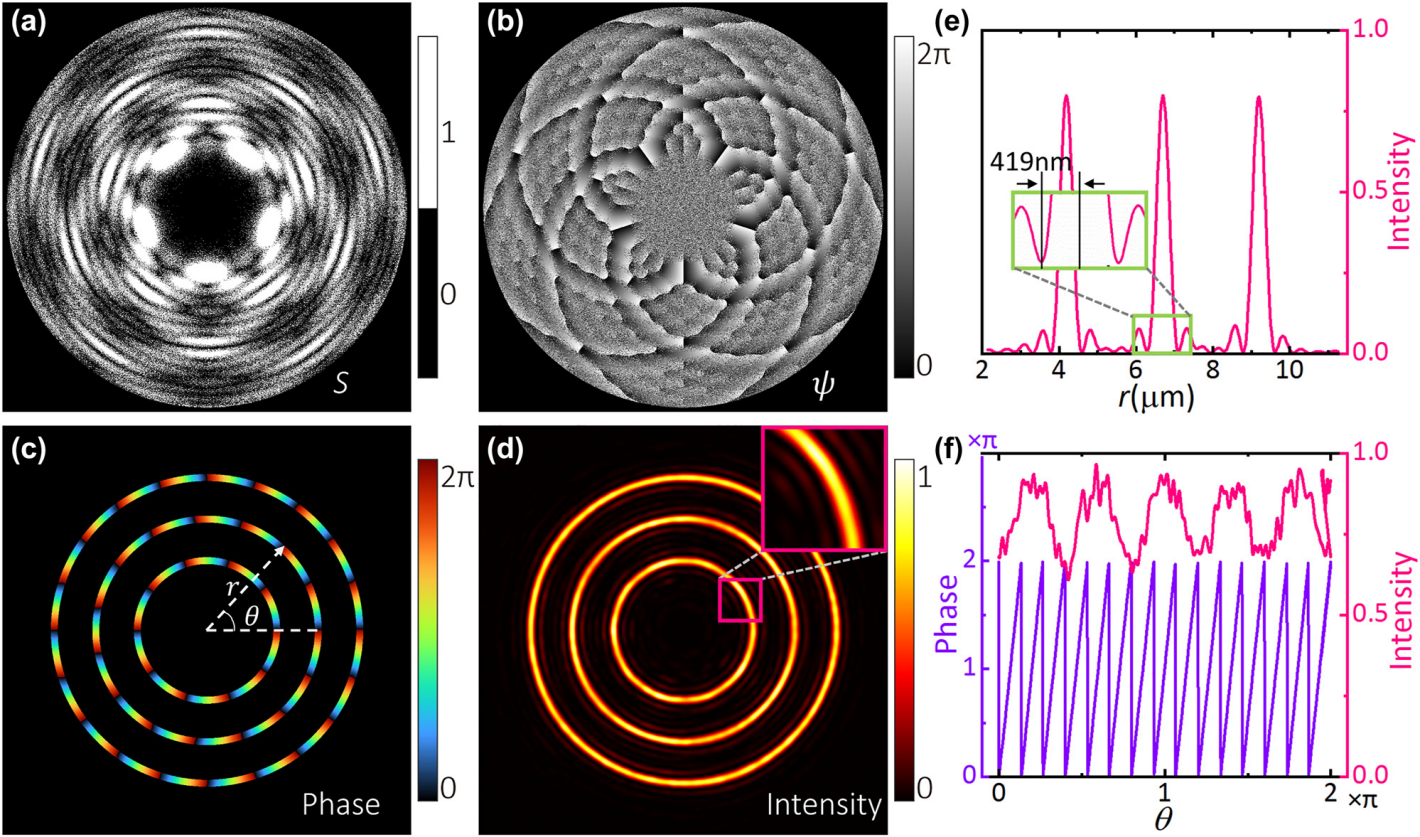
Numerical results of a CROT consisting of three rings with the radii *R* = 4.2, 6.7, 9.2 μm and the topological charges *l* = 10, 15, 20, respectively, and having *β* = 2.2. (a) Shape function *S*. (b) Hologram *ψ*. (c–d) Phase and intensity of the CROT. The inset in (d) is the magnification of part of the inner ring and shows some of the annular sidelobes. (e) Azimuth angle-averaged intensity versus radius *r*. The width of the middle ring is ∼419 nm equal to the diffraction limit *δ*. (f) The phase and intensity profiles of the middle ring along the azimuthal direction.

Denote the field’s magnitude and phase by

(5) $$\begin{array}{c} \begin{array}{c} A=\left|E\left(\rho ,\varphi \right)\right|,\;\;\;\;\;\phi =\arg\left(E\left(\rho ,\varphi \right)\right) \end{array}, \end{array}$$

where |∗| means taking the modulus and arg(*) means taking the phase. Typically, the magnitude *A* varies with spatial coordinates. Since most of the commercially available liquid crystal spatial light modulators (SLMs) are pure phase-type, it is of practice to convert the complex amplitude information to a phase-only one. An approach is to take the phase *ϕ* as a hologram and address it on the SLM. This method is straightforward, but the reconstructed target has significant sidelobes due to the discarded amplitude information. Another approach is the complex amplitude modulation technique [34], which simultaneously encodes arbitrary amplitude and phase into pure phase. Clark et al. [35] summarized the mathematics of several complex amplitude encoding methods and compared their generation quality and diffraction efficiency. These methods have advantages and disadvantages in terms of peak signal-to-noise ratio and diffraction efficiency, and some have complex mathematical calculations for generating holograms. Fortunately, Roichman et al. [36] previously proposed a computationally simple and highly diffraction-efficient method known as shape-phase holography. We employ this complex amplitude encoding technique here. The key to the method is to use the amplitude *A* to generate the shape function *S*, a binary mask taking the value 0 or 1. For the field given by Eq. (5), the shape function *S* is as follows

(6) $$\begin{array}{c} S\left(\rho ,\varphi \right)=\left\{\begin{array}{cc} 1,\; & rand\left(0,1\right)\leq \beta \frac{A\left(\rho ,\varphi \right)}{\max\left(A\right)}\\ 0,\; & r\;and\;\left(0,1\right)>\beta \frac{A\left(\rho ,\varphi \right)}{\max\left(A\right)} \end{array}\right., \end{array}$$

where rand(0,1) is a uniform random number between 0 and 1, max(*) means taking the maximum value, and the parameter *β* is a control factor. With the shape function *S*, the phase-only hologram takes the form:

(7) $$\begin{array}{c} \psi =S\phi +\left(1-S\right)\phi_{R}, \end{array}$$

where *ϕ*_*R*_ is a random phase. The shape function *S* divides the whole hologram into valid (*S* = 1) and invalid (*S* = 0) regions. The valid region retains the phase of the complex amplitude *E*(*ρ*, *φ*), while the invalid region is filled using a random phase. Using the shape-phase holography Eqs. (6) and (7), CROTs are to be generated. To match our experimental system, the light wavelength *λ* = 1064 nm and the focal length *f* = 200/60 mm are chosen in simulations; a water-immersion objective (Apo Plan IR, 60×/NA1.27, Nikon Corp.) is used there with the diffraction limit *δ* = 0.5*λ*/NA = 419 nm. Figure 1 illustrates a CROT containing three rings with the radii *R* = 4.2, 6.7, 9.2 μm and the topological charges *l* = 10, 15, 20, respectively, and having *β* = 2.2. We will discuss determining the value of the parameter *β* in the next section. Figure 1(a) presents the shape function *S* obtained from Eq. (6), Figure 1(b) is the phase-only hologram *ψ* obtained from Eq. (7) and Figure 1(c and d) show the phase and intensity of the generated CROT, respectively. After averaging over the azimuth angle, the intensity versus radius *r* is shown in Figure 1(e). We define the width of a ring as the distance from the ring peak to the first zero. Here, the width of a typical ring (middle ring) is ∼419 nm equal to the diffraction limit *δ*. The phase and intensity profiles of the middle ring in the azimuthal direction are shown in Figure 1(f). It can be seen that the phase has 15 periods, and the intensity has a mean value of 0.799 with a standard deviation of 0.088.

### 2.2 Evaluation of CROT quality

The results presented in Figure 1 show that the field of CROT generated by using the complex amplitude encoding technique is flawed to some extent, e.g., the annular sidelobes are relatively strong and the intensity is not homogeneous along the azimuthal direction. In order to evaluate the quality of the generated CROTs, we next define three evaluation parameters: energy ratio, uniformity, and diffraction efficiency.

#### 2.2.1 Energy ratio

The energy ratio is defined to be the ration of the energy contained in all the annular regions to the total energy of the light field. Write *I*(*r*, *θ*) = |*E*(*r*, *θ*)|^2^, the energy (per unit distance) contained in the *n*th annular region is thus

(8) $$\begin{array}{c} V_{n}=\int_{0}^{2\pi }\int_{R_{n}-\delta }^{R_{n}+\delta }I\left(r,\theta \right)rdrd\theta , \end{array}$$

where *δ* = 0.5*λ*/NA is the diffraction limit and the *n*th annular region is given by (*R*_*n*_ − *δ*) < *r* < (*R*_*n*_ + *δ*). The total energy passing through the focal plane is

(9) $$\begin{array}{c} V=\int_{0}^{2\pi }\int_{0}^{+\infty }I\left(r,\theta \right)rdrd\theta \cdot \end{array}$$

The energy ratio can be expressed as

(10) $$\begin{array}{c} r_{e}=\frac{1}{V}\overset{N}{\underset{n=1}{\sum }}V_{n}, \end{array}$$

where *N* denotes the total number of rings. The larger *r*_*e*_, the fewer sidelobes the rings have.

#### 2.2.2 Uniformity

The uniformity describes the fluctuation of the peak intensities of rings with azimuth angle. The uniformity of the *n*th ring can be expressed as

(11) $$\begin{array}{c} u_{n}=1-\frac{\max\left(I_{n}\right)-\min\left(I_{n}\right)}{\max\left(I_{n}\right)+\min\left(I_{n}\right)}, \end{array}$$

where *I*_*n*_ = *I*(*R*_*n*_, *θ*) is the peak intensity of the *n*th ring, max(*) and min(*) denote taking the maximum and minimum values, respectively. Different rings have different uniformity *u*_*n*_, and we use the minimum value of *u*_*n*_ to represent the overall uniformity. The uniformity of CROT can thus be expressed as

(12) $$\begin{array}{c} u=\min\left(u_{n}\right). \end{array}$$

The larger *u*, the fewer “hot spots” the rings have.

#### 2.2.3 Diffraction efficiency

Here, the diffraction efficiency is defined as the ratio of the energy in all the annular regions to the energy incident on the hologram. For the hologram obtained by Eq. (7), the light from the invalid region will be blocked by the optical system. As a result, the diffraction efficiency can be expressed as the product of the proportion of the valid region and the energy ratio:

(13) $$\begin{array}{ll} e & =\frac{1}{M}\overset{M}{\underset{j=1}{\sum }}S_{j}\times \frac{1}{V}\overset{N}{\underset{n=1}{\sum }}V_{n}\\ & =\frac{1}{M}\overset{M}{\underset{j=1}{\sum }}S_{j}\times r_{e}, \end{array}$$

where *S*_*j*_ is the *j*th element value of the shape function *S*, and *M* is the total number of elements. The larger *e*, the more efficient the energy utilization is.

**Figure 2:**
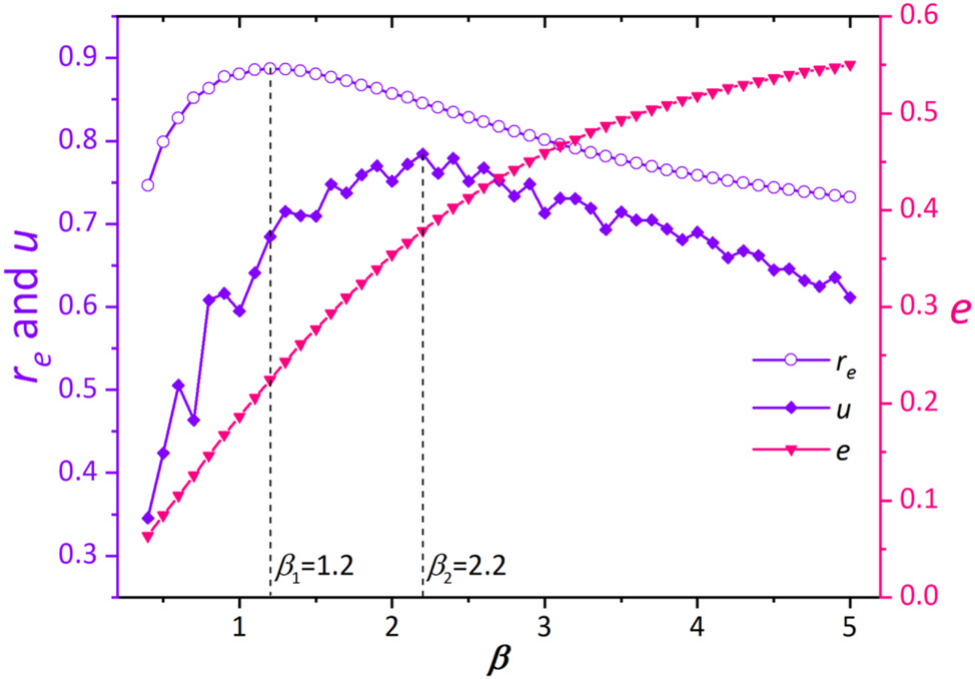
Curves of the energy ratio *r*_*e*_, uniformity *u* and diffraction efficiency *e* as a function of *β*. The simulated CROT has *R* = 4.2, 6.7, 9.2 μm and *l* = 10, 15, 20, respectively.

Consider again the CROT (*R* = 4.2, 6.7, 9.2 μm, *l* = 10, 15, 20) shown in Figure 1. We plot the curves of *r*_*e*_, *u*, and *e* versus *β* in Figure 2. As demonstrated, *r*_*e*_ reaches a maximum value of 0.89 at *β*_1_ = 1.2, *u* reaches a maximum value of 0.78 at *β*_2_ = 2.2, and *e* increases monotonically with *β*. In optical trapping, the uniformity of CROT can affect the rotation of particles and low uniformity might lead particles to stop rotating. As far as the uniformity is concerned, it is adequate to take *β*_2_ = 2.2 as the optimal value at which the values of *r*_*e*_, *u*, and *e* are 0.85, 0.78, and 0.38, respectively. For CROTs with other radii and topological charges, the optimal value of *β* may vary from 1 to 3.

In particular, it is noted that the Bessel function *J*_*l*_(*) in Eq. (4) is defined in [0,+∞], whereas the hologram contains only a limited center region. The truncation of the Bessel function leads to a slight difference among the average intensities of rings. However, the difference can be corrected by applying additional weights *w*_*n*_ to Eq. (4). The weights *w*_*n*_ are generally close to 1, with little variation among them. For the example shown in Figure 1, the weights are 1.000, 1.035 and 1.010. When generating a phase-only hologram, we first determine the optimal value of the parameter *β*, and then finely adjust Eq. (4) by applying the weights *w*_*n*_ if the equal average intensities of different rings are required.

### 2.3 Optical trapping device

**Figure 3:**
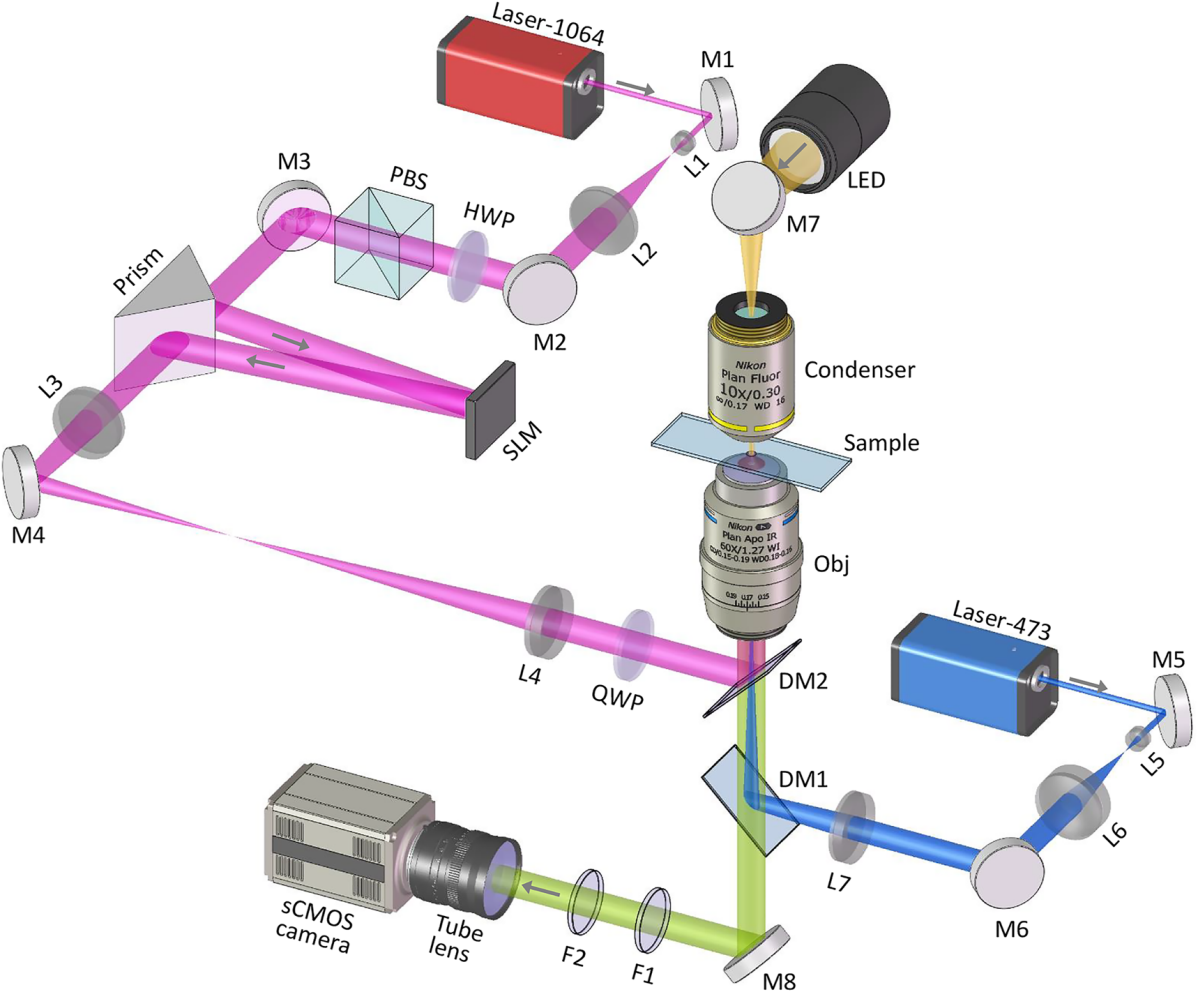
Schematic diagram of the experimental setup. The trapping optical path is shown in red, where the SLM modulates the incident 1064-nm beam to generate optical traps. A 473-nm laser and an LED light source are used for fluorescence imaging and bright-field imaging, respectively. The imaging optical path is green, where the tube lens has a focal length of 300 mm and is connected to a 1.8 × magnification lens, resulting in ∼160 × magnification. M, mirror; L, lens (*f*1 = 10, *f*2 = 100, *f*3 = 150, *f*4 = 150, *f*5 = 10, *f*6 = 50, *f*7 = 150 mm); HWP, half-wave plate; QWP, quarter-wave plate; PBS, polarizing beam splitter; SLM, phase-only liquid crystal spatial light modulator; DM, dichroic mirror; Obj, objective lens; F, filter.

The experimental setup is shown in Figure 3. The beam (*λ* = 1064 nm) from a fiber laser (5000 mW; VFLS-1064-B-SF-HP, Connet Laser Technology Co., Ltd., China) is expanded and collimated by a telescope (L1 and L2). A polarizing beam splitter (PBS) and a half-wave plate (HWP) are used to filter the horizontally polarized light. A phase-only liquid crystal spatial light modulator (SLM; 1920 × 1080 pixels, 8 μm pixel pitch; PLUTO-2-NIR-049, Holoeye Photonics AG, Germany) modulates the incident beam by addressing a phase hologram computed with the above described method. An isosceles prism is placed ∼100 mm in front of the SLM panel to shorten the optical path. A 4-*f* system consisting of lenses L3 and L4 relays the SLM plane to the back focal plane of the objective lens (Apo Plan IR, 60×/NA1.27, Water Immersion, Nikon Corp., Japan). A quarter-wave plate (QWP) converts the linear polarization to circular polarization. The sample slide is placed on a motorized XY translation stage (PZ-2000FT, Applied Scientific Instrumentation Inc., USA) and a piezoelectric *Z*-axis stage (PZ-2500FT, Applied Scientific Instrumentation Inc., USA). The system can perform fluorescence imaging and bright-field imaging. A 473-nm laser (500 mW; gem 473, Laser Quantum Ltd., UK) is expanded and collimated by a telescope (L5 and L6) and then used to excite the sample for fluorescence imaging. An LED light source (MCWHL7, Thorlabs Inc., USA) illuminates the sample after passing through a condenser for bright-field imaging. Here, the condenser is an objective (Plan Fluor, 10×/NA0.30, Nikon Corp., Japan) with a 16 mm working distance. Dichroic mirrors DM1 (Di03-R488/561-t1-25x36, Semrock Inc., USA) and DM2 (Di02-R1064-25x36, Semrock Inc., USA) are used to combine or separate beams of different wavelengths. Filters F1 (FF01-770/SP-25, Semrock Inc., USA) and F2 (FF01-523/610-25, Semrock Inc., USA) are used to filter out the trapping beam (1064 nm) and excitation beam (473 nm), respectively. An sCOMS camera (2048 × 2048 pixels, 6.5 μm pixel pitch; ORCA-Flash 4.0 V2 C11440-22CU, Hamamatsu Photonics K.K., Japan) records the images of samples. The tube lens has a focal length of 300 mm and is connected to a 1.8 × magnification lens, resulting in ∼160 × magnification of the imaging optical path.

The aberration of the system can be fitted with Zernike polynomials and corrected using the SLM. The shape of a LG mode with topological charge of +1 is taken as an optimization goal, and the weights of the Zernike polynomials are finely tuned to fit the aberration of the system. After obtaining the aberration correction hologram, the SLM is addressed with it so that the aberration of the system can be corrected. Under the condition of SLM displaying a blank pattern, the ratio of the light power at the entry pupil of the objective to the output power of the laser is 0.65. The first-order diffraction efficiency of the SLM is 0.93 provided by the manufacturer. The separation of the zero- and first-order diffracted beams is achieved by addressing a blazed grating on the SLM. A silver-plated mirror placed on the focal plane of the objective is used to reflect the 1064-nm laser to measure the intensity profiles of CROTs.

Sample chambers with a depth of approximately 20 μm are made with slides, coverslips (thickness 0.17 mm), double-sided tape, and sealed with glue after injection of a suspension containing particles. Polystyrene (PS) fluorescent microspheres (FluoSpheres Carboxylate 1.0 μm Yellow-green 505/515 nm, Thermo Fisher Scientific Inc., USA) and gold (Au) particles (Gold Nanoparticles 1000 nm, Beijing Biotyscience Technology Co., Ltd., China) are used for the experiments, respectively. At the initial step of the experiment, a micro-ruler is used to calibrate the pixel size of acquired images.

## 3 Results

### 3.1 Independent control of topological charges

**Figure 4:**
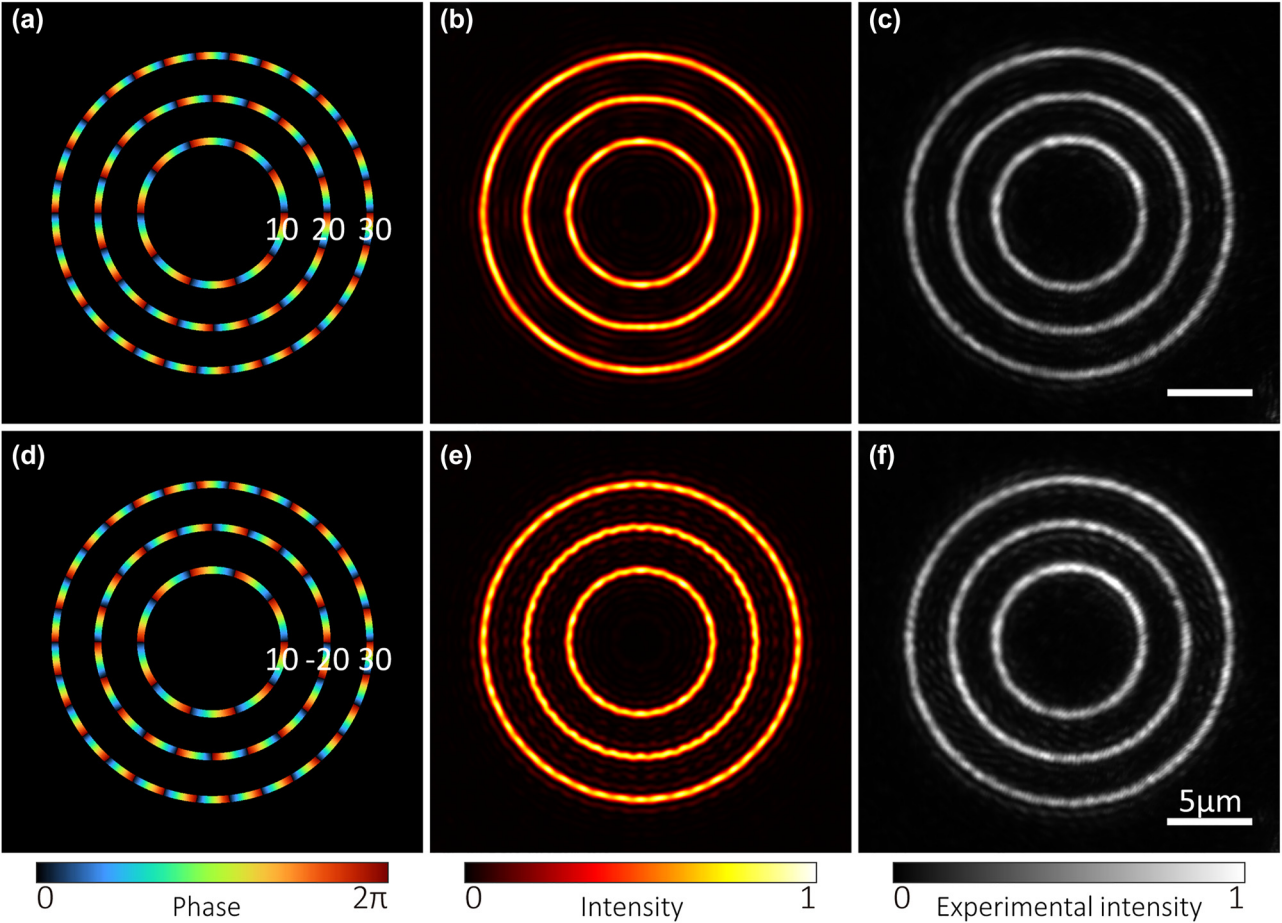
Independent control of topological charges of rings. (a, b) Simulated phase and intensity profiles of a CROT (*R* = 4.2, 6.7, 9.2 μm, *l* = 10, 20, 30). (c) Experimentally measured intensity profile corresponding to (b). (d, e) Simulated phase and intensity profiles of a CROT (*R* = 4.2, 6.7, 9.2 μm, *l* = 10, −20, 30). Topological charge of the middle ring in (d) is opposite to that of (a). (f) Experimentally measured intensity profile corresponding to (e). The scale bar is 5 μm.

Conventional vortex beams (e.g., LG beams) have a topological charge-dependent ring radius, while the radii of rings in the CROT are independent of the topological charges. Figure 4(a–c) demonstrate a CROT of three rings with respective radii *R* = 4.2, 6.7, 9.2 μm and respective topological charges *l* = 10, 20, 30. Here, *β* = 1.7, the weights are 1.000, 1.114, and 1.118, and the three evaluation parameters *r*_*e*_, *u*, and *e*, are 0.87, 0.78, and 0.40, respectively. The phase in Figure 4(a) exhibits a periodic change along the azimuthal direction with respective periods of 10, 20, 30 in order from inside to outside, in agreement with the predesigned topological charges. Figure 4(b and c) show the theoretical and experimentally measured intensity profiles, respectively. A blazed grating is utilized in the experiment to separate the unwanted zero-order spot. Figure 4(d–f) show the results for a CROT with the topological charge of the middle ring changed from 20 to −20; other parameters are *β* = 2.1, the weights are 1.000, 1.150, and 1.160, and *r*_*e*_, *u*, and *e*, are 0.85, 0.79, and 0.48, respectively.

When the topological charge of the middle ring changes from 20 to −20, *r*_*e*_ becomes smaller, therefore sidelobes become stronger. However, *u* changes little, so the uniformity does not change noticeably. The reason for the stronger variation of sidelobes as the topological charge of the middle ring changes from 20 to −20 is due to the interference among sidelobes. The sidelobes show interference patterns in the inter-ring region, exhibiting a periodic distribution along the azimuthal direction with the number of periods equal to |*l*_1_ − *l*_2_|, where *l*_1_ and *l*_2_ are the topological charges of the neighboring rings. For the CROT with *l* = 10, 20, 30, the number of periods in sidelobes is equal to |20–10| = |20–30| = 10, while for the CROT with *l* = 10, −20, 30, the number of periods is |−20–10| = 30 and |−20–30| = 50, respectively. The growing number of periods indicates that the sidelobes present a more rapid change. Thus, the CROT with *l* = 10, −20, 30 has more obvious sidelobes and stronger appearance in regions with large radii.

**Figure 5:**
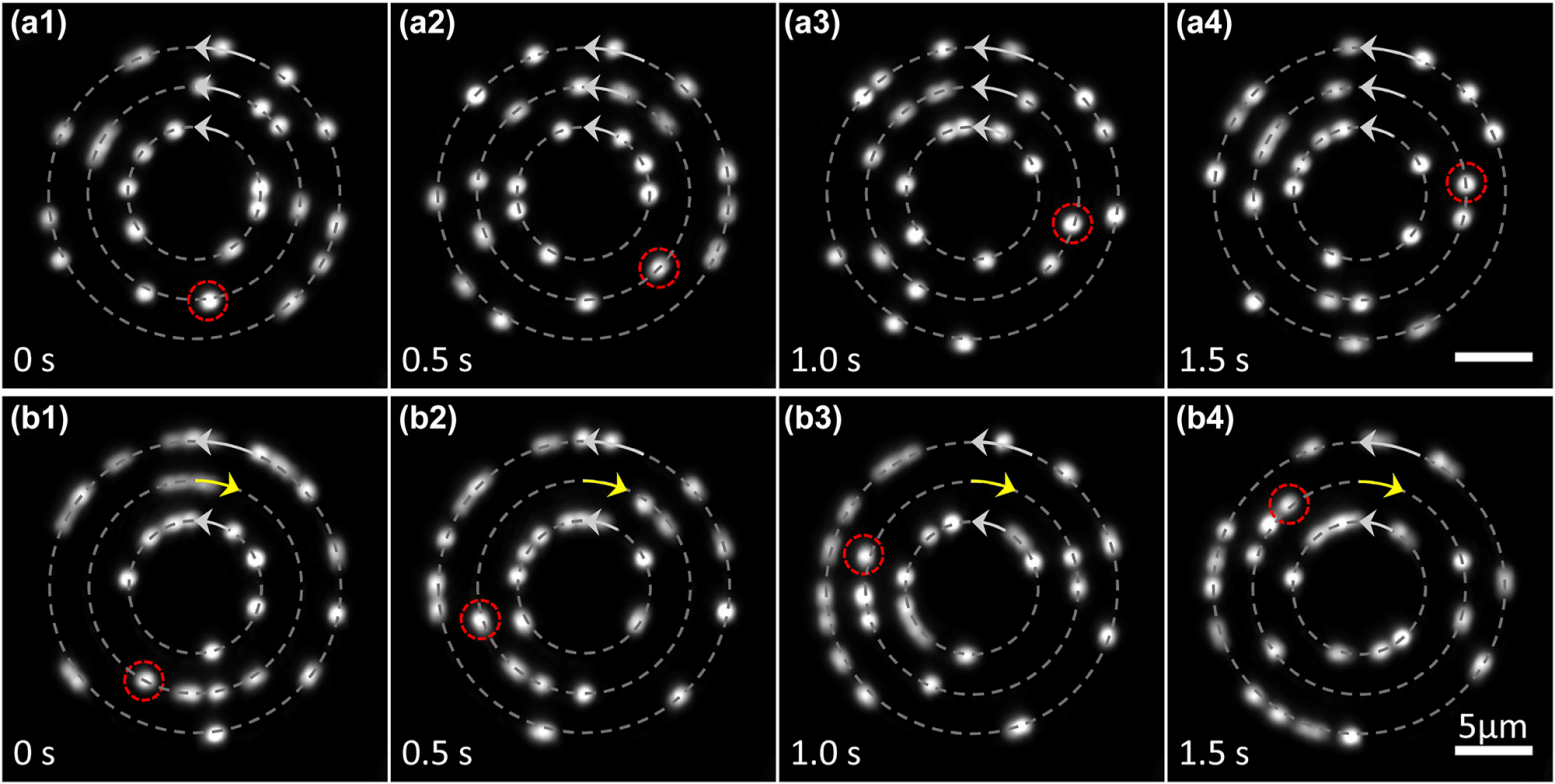
Manipulation of fluorescent PS microspheres with CROTs. (a) CROT (*R* = 4.2, 6.7, 9.2 μm, *l* = 10, 20, 30) drives the PS microspheres to rotate. The direction of motion of all microspheres is counterclockwise. (b) Another CROT (*R* = 4.2, 6.7, 9.2 μm, *l* = 10, −20, 30) manipulates PS microspheres. The direction of motion of the microspheres on the middle ring is clockwise. The gray dashed circles mark the positions of rings, and the arrows indicate the directions of motion of the microspheres. The red dashed circles mark the positions of a specific microsphere at different times. The total light power on the three rings is about 500 mW. The diameter of the microspheres is 1 μm, and the scale bar is 5 μm. The dynamic rotations of microspheres are recorded in Visualization 1 and Visualization 2 in the Supplementary Material.

The trapping of PS microspheres (1 μm in diameter and 1.590 in refractive index) with CROTs is shown in Figure 5. The illumination wavelength is 473 nm, suitable for fluorescence imaging. Dielectric particles such as PS microspheres are trapped at the location of local intensity maximum, i.e., on the rings. In the presence of azimuthal phase gradient, the particles rotate in the gradient direction, i.e., the azimuthal direction. In Figure 5(a1–a4), the CROT is a three-ring structure with *R* = 4.2, 6.7, 9.2 μm and *l* = 10, 20, 30, respectively, and we see that the PS microspheres on all three rings move counterclockwise. In Figure 5(b1–b4), we reverse the sign of the topological charge of the middle ring, and the PS microspheres on the middle ring are observed to rotate clockwise, as desired.

### 3.2 Tunable DR-POV

**Figure 6:**
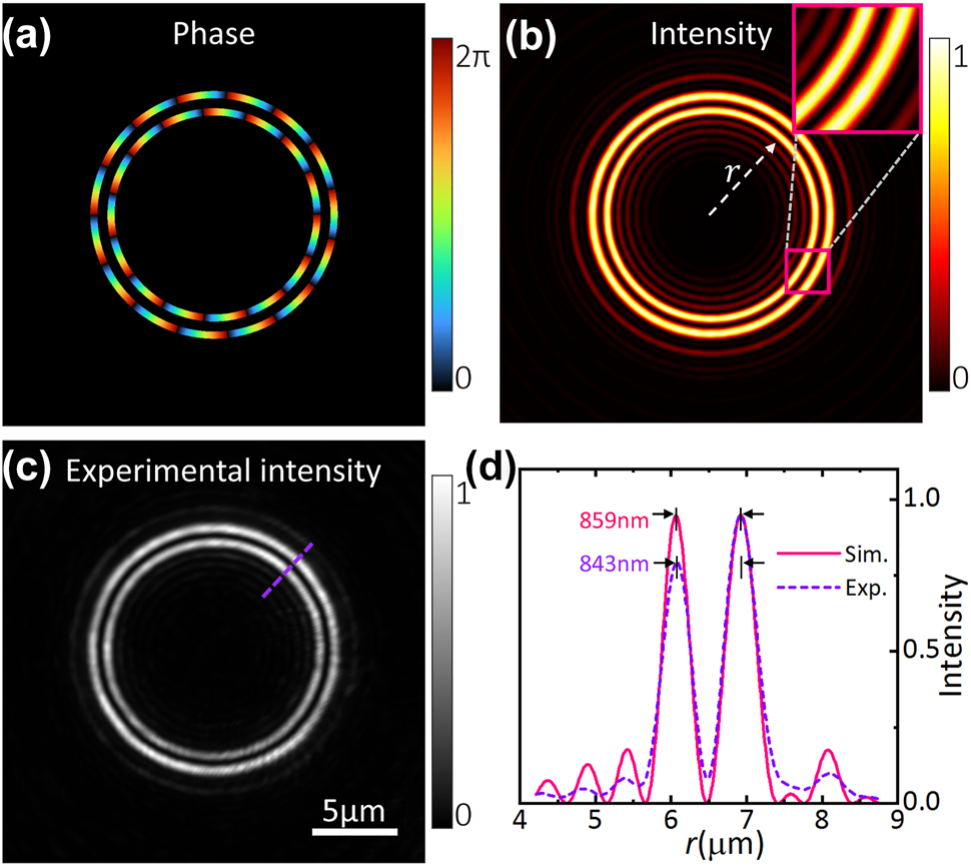
Tunable DR-POV (*R*_1_ = 6 and *R*_2_ = 7 μm, *l*_1_ = 15 = *l*_2_, and initial phase difference π). (a, b) Simulated phase and intensity profiles. Here, the *β* = 4.8, the weights are 1.000 and 0.921, and *r*_*e*_, *u*, and *e* are 0.78, 0.94, and 0.52, respectively. (c) Experimentally measured intensity profile corresponding to (b). (d) Azimuth angle-averaged intensity versus radius *r*. The scale bar is 5 μm.

As a special case of CROTs, a tunable double-ring POV (DR-POV) can be generated by combining two rings with the parameters *l*_1_ = *l*_2_ and *R*_1_ ≈ *R*_2_. Since the two rings have almost identical radii, an initial phase difference π is added to distinguish them. Here, the tunability refers to the capability of adjustable ring-peak distance. Figure 6 shows a DR-POV with *R*_1_ = 6 and *R*_2_ = 7 μm and *l*_1_ = 15 = *l*_2_. The theoretical and experimental values for the ring-peak distance are 859 nm and 843 nm, respectively, approximately equal to 2*δ*. As shown in Figure 6(b), the DR-POV has strong sidelobes, the root cause of which is that the complex amplitude coding method loses some of the information. An ideal single-ring POV is a ring without sidelobes, and its spectrum corresponds to a higher-order Bessel function, which is a complex amplitude. But the process of encoding the complex amplitude into a phase-only hologram loses some of the information, resulting in a reconstructed POV with sidelobes. In the example of DR-POV, each of the two rings has sidelobes. They interfere with each other due to their very close radii, generating the strong sidelobes. We set the parameter *β* = 4.8 to obtain maximum uniformity (*u* = 0.94). However, the larger *β* leads to a lower energy ratio (*r*_*e*_ = 0.78), which also means stronger sidelobes.

Tunable DR-POVs have two parameters, *R*_1_ and *R*_2_, to determine the value of the ring-peak distance. It is noted that the ring-peak distance is variable down to a minimum of approximately *δ*. Although previously reported DR-POVs generated by using circular Dammann gratings or polarization modulation [29–31] have a ring-peak distance of ∼*δ*, our DR-POVs have a modifiable ring-peak distance, hence being able to match the particle’s size when trapping metal particles.

**Figure 7:**
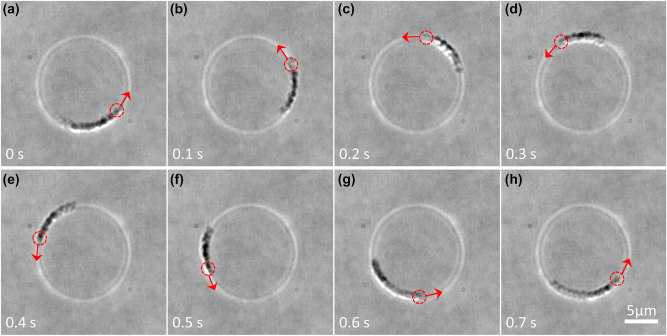
Manipulation of Au particles with tunable DR-POV. (a–h) Tunable DR-POV (*R*_1_ = 6 and *R*_2_ = 7 μm, *l*_1_ = 15 = *l*_2_) drives the Au particles to rotate counterclockwise. The bright rings come from the 1064-nm laser reflected from the glass-water interface and show the position of the double-ring optical trap. The red dashed circles mark the positions of a specific particle at different times. The red arrows mark the direction of motion of that particle. The total light power on the double rings is about 250 mW. The particle diameter is about 1 μm and the scale bar is 5 μm. The dynamic rotation of particles is recorded in Visualization 3 in the Supplementary Material.

In Figure 7, we use a tunable DR-POV to manipulate metal particles (Au particles of diameter 1 μm), which are trapped in the dark region between two bright rings due to a strong absorption. Since the Au particles do not emit fluoresce, an LED light source is used for the bright-field imaging. The ring-peak distance in the experiment is ∼843 nm, slightly smaller than the diameter of Au particles. The trapped Au particles are seen to rotate counterclockwise. The particles are pushed up against the slide to equalize the axial scattering force.

**Figure 8:**
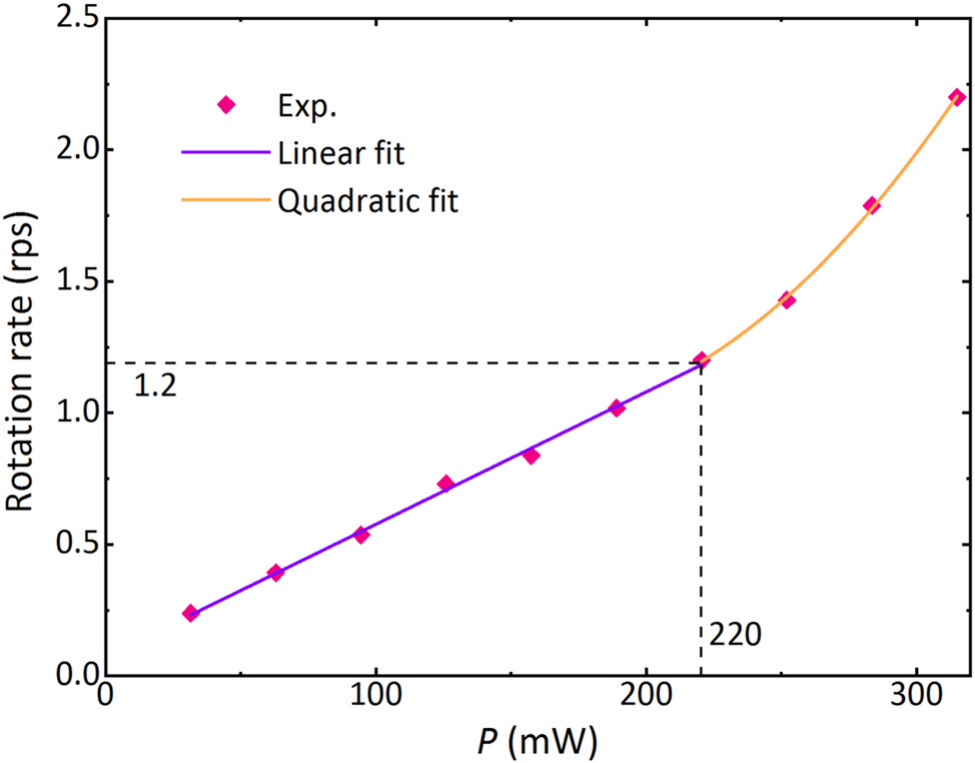
The dependence of the rotation rate of Au particles on the light power of the double rings. When the light power *P* ≤ 220 mW, the rotation rate is linearly related to *P*. However, when *P* > 220 mW, the rotation rate is quadratically polynomial with *P*. Dynamic rotations are recorded at each light power for 30 s, and then the rotation rates are calculated.

We define the rotation rate as the number of circles per second of the Au particles rotating around the center of rings. With increasing the light power on the double rings, the rotation rate of the Au particles increases. Figure 8 shows the rotation rate versus the light power. When the light power *P* ≤ 220 mW, the rotation rate is linearly related to *P*. However, when *P* > 220 mW, the rotation rate is quadratically polynomial with *P*. Such a nonlinear dependence may result from the photophoretic force generated by the photothermal effect [37].

## 4 Discussion and conclusion

We have shown a method for the generation of CROTs, which has the following distinctive features. First, CROTs expand the concept of POVs by increasing the number of rings. No longer limited to single or double rings, CROTs can consist of any number of rings in principle. Second, the complex amplitude coding method is improved by introducing a parameter *β*, which can maximize the uniformity of CROTs. Last, a holographic optical tweezers setup was built to demonstrate the practical application of CROTs by conducting optical trapping and various orbital rotations of PS microspheres and Au particles. The main advantage of CROTs is the manipulation capability of parallel orbital rotations. The topological charge is independent of the radius and can be controlled individually for each ring, thus allowing parallel rotations with customized directions and speeds. Another merit is that the method is simple and straightforward of obtaining the phase holograms of CROTs. The holograms are generated by the superposition of higher-order Bessel functions followed by a complex amplitude encoding, which is more direct and faster than the iterative method. Nevertheless, to get a perfect CROT, there are some challenges in terms of sidelobes, uniformity and diffraction efficiency. For continuous orbital rotation of particles, the uniformity is the primary concern. The maximum uniformity is achieved by virtue of the parameter *β*. The interference of sidelobes with rings could degrade the uniformity, but this negative effect could be weakened by appropriately increasing the ring-peak distance. The diffraction efficiency can be considerably improved by choosing an appropriate complex amplitude encoding method, such as the shape-phase holography.

It is worth noting that the topological charge-independent property of the CROT radius is consistent with that of a single-ring POV or a double-ring POV, because each ring in a CROT is a POV. However, constrained by the finite aperture of the microscope objective, a ring cannot be infinitely narrow as described by Eq. (3). A ring has a diffraction-limited focusing in the radial direction with a width equal to 0.5*λ*/NA, and has an approximately uniform intensity in the azimuthal direction with a phase gradient. Additionally, it is also diffraction-limited focusing in the *Z*-axis direction with an intensity profile similar to that of a Gaussian point trap. The diffraction limit in the Z-axis direction is *λ*/[*n*(1 − cos*α*)], which is roughly three times as large as the lateral one. Achieving a three-dimensional (3D) optical trap is challenging because the axial diffraction limit is much larger than the transverse one, resulting in an imbalance between the *Z*-axis optical force and the gravity of a particle. In this paper, we have only demonstrated the trapping and orbital rotation of PS fluorescent microspheres by CROTs in the focal plane, and future studies will explore their capabilities in 3D trapping. Theoretically, there is no limit to the number of rings in a CROT, but more rings mean more energy required for optical trapping. Here, we demonstrate the manipulation of PS fluorescent microspheres by CROTs with three rings.

As a special case of CROTs, a tunable DR-POV has been used to manipulate Au particles. Previously reported DR-POVs generated by using circular Dammann gratings or polarization modulation have a ring-peak distance of about the diffraction limit. Here, the tunable DR-POV has a similar structure, but the ring-peak distance is modifiable. Such flexibility makes it ideal for manipulating particles ranging in size from submicrometers to several micrometers. Consider another special case of CROTs in which two rings have parameters *R*_1_ = *R*_2_ and *l*_1_ ≠ *l*_2_, the rings will overlap and interfere. The interference pattern will show periodic petals in the azimuthal direction with the number of periods equal to |*l*_1_ − *l*_2_|. This petal structure allows the localization of particles without inducing continuous rotation. In addition, a tunable triple-ring POV can be generated by simply modifying parameters. We show only a tunable DR-POV as an example.

The motion of particles in a liquid environment is retarded by viscous forces, and the rotation rate is related to the viscosity of the fluid. Rotational optical tweezers have been used to measure intracellular viscosity [38]. In addition, the orbital rotation of trapped particles can be utilized to construct biocompatible microviscosity sensors [39]. As tools to study the orbital rotation of trapped particles, CROTs will be useful in microfluidic viscosity measurements.

In summary, our proposed CROTs are characterized by high quality, diffraction-limited focusing, and topological charge-independent size. Its applications in optical trapping have been experimentally demonstrated by several examples, including the manipulation of PS fluorescent microspheres and Au particles. As a novel tool, it is well suited to study the orbital rotation of trapped particles. We expect more potential applications in other areas, such as microfluidic viscosity measurement, laser micro-nano fabrication, and OAM multiplexed optical communication.
